# Impact of interorganelle coordination between the conventional early secretory pathway and autophagy in cellular homeostasis and stress response

**DOI:** 10.3389/fcell.2023.1069256

**Published:** 2023-04-21

**Authors:** Diego Tapia, Viviana A. Cavieres, Patricia V. Burgos, Jorge Cancino

**Affiliations:** ^1^ Cell Biology of Interorganelle Signaling Laboratory, Centro de Biología Celular y Biomedicina (CEBICEM), Facultad de Medicina y Ciencia, Universidad San Sebastián, Santiago, Chile; ^2^ Organelle Phagy Lab, Centro de Biología Celular y Biomedicina (CEBICEM), Facultad de Medicina y Ciencia, Universidad San Sebastián, Santiago, Chile; ^3^ Centro Ciencia & Vida, Fundación Ciencia & Vida, Santiago, Chile

**Keywords:** endoplasmic reticulum (ER), KDEL receptor (KDELR), lipid droplets (LD), protein kinase A (PKA), plasma membrane (PM), traffic-induced degradation response for secretion (TIDeRS)

## Abstract

The conventional early secretory pathway and autophagy are two essential interconnected cellular processes that are crucial for maintaining cellular homeostasis. The conventional secretory pathway is an anabolic cellular process synthesizing and delivering proteins to distinct locations, including different organelles, the plasma membrane, and the extracellular media. On the other hand, autophagy is a catabolic cellular process that engulfs damaged organelles and aberrant cytosolic constituents into the double autophagosome membrane. After fusion with the lysosome and autolysosome formation, this process triggers digestion and recycling. A growing list of evidence indicates that these anabolic and catabolic processes are mutually regulated. While knowledge about the molecular actors involved in the coordination and functional cooperation between these two processes has increased over time, the mechanisms are still poorly understood. This review article summarized and discussed the most relevant evidence about the key molecular players implicated in the interorganelle crosstalk between the early secretory pathway and autophagy under normal and stressful conditions.

## Introduction

The secretory pathway is responsible for synthesizing, folding, sorting, and delivering a variety of cellular proteins to distinct locations, such as different organelles, the plasma membrane, and the extracellular media. In the early secretory pathway, the endoplasmic reticulum (ER) ensures the correct entry of nascent proteins safeguarding their correct post-translational modifications and folding. Folded proteins are incorporated into ER-derived transport vesicles where they are delivered to the Golgi apparatus, the next station of the early anterograde transport pathway. On the other hand, ER-resident proteins that escape from the ER and cycle between the two early stations, need to constantly return from the Golgi to the ER, a process called the retrograde transport pathway (Barlowe and Miller, 2013). Both anterograde and retrograde early secretory pathways are critical to maintaining organelle structure, function, and cell homeostasis (Farhan et al., 2008; Cancino et al., 2014; Hanna et al., 2018).

Meanwhile, macroautophagy (hereafter referred to as autophagy) is a catabolic cellular process that engulfs damaged organelles and aberrant cytosolic components into a double membrane called the autophagosome. The autophagosome then fuses with the lysosomes to form a hybrid organelle called an autolysosome, which digests and recycles the molecular components (Mizushima et al., 2008; Pu et al., 2016; Mizushima, 2018). The process of forming an autophagosome begins with the formation of a pre-autophagosomal structure (PAS). The PAS initiates the nucleation of essential components that are necessary for the elongation, maturation, and sealing of the double membrane that forms the autophagosome (Kawamata et al., 2008; Suzuki and Ohsumi, 2010; Harada et al., 2019). The autophagosomes then fuse with lysosomes to form an autolysosome (Reggiori and Ungermann, 2017).

It is important to note that there is a mutual regulation between the early conventional secretory pathway and autophagy (summarized in Table 1). Recent research suggests that the early secretory pathway, including de ER and Golgi apparatus, plays an important role in the regulation of autophagy beyond providing membrane sources for autophagosome formation. Conversely, autophagy regulates important organelles and machinery of the early secretory pathways, such as the ER size through ER-phagy (Gubas and Dikic, 2022), turnover of the Golgi apparatus (Nthiga et al., 2021; Rahman et al., 2022), and the quantity and efficiency of the quality control machinery that defines efficient protein secretion, such as lectin binding proteins that recognize unfolded proteins for degradation (Park et al., 2014) and the proteasome through proteophagy (Albornoz et al., 2019; Quinet et al., 2020). Moreover, membrane composition alters both, the early secretory pathway, and autophagy in yeast (Li et al., 2020). The disruption of triacylglycerol synthesis led to diacylglycerol (DAG) accumulation disturbing the balance of ER-Golgi protein trafficking, resulting in ER swelling, loss of the Golgi apparatus, and autophagy inhibition.

**TABLE 1:** Common regulators of the ER-Golgi early secretory pathway and autophagy. The main proteins described in this work were summarized in this table describing the role of those protein complexes and regulators on both, the secretory and autophagy pathways.

*Involved on ER-Golgi anterograde and retrograde membrane transport. Bethune and Wieland (2018)	COPI and COPII	*Genetic defects in COPII machinery impaired the autophagosomes formation Ishihara et al. (2001)
*Essential role at ERES subdomains where proteins are sorted into the secretory system Kurokawa and Nakano (2019)		*Autophagosome nucleation, elongation and maturation facilitating the assembly of lipidation machinery and promoting LC3 lipidation Karanasios et al. (2016)
Monomeric GTPases that control protein and membrane transport from the ER to the Golgi Mizuno-Yamasaki et al. (2012)	sar1/Rab	Silencing of either, Sari or Rab1 (which negatively affect membrane transport at ER level) impair autophagosome biogenesis, as determined by the lack of LC3 recruitment and LC3 lipidation Zoppino et al. (2010)
PI3K are required for the formation of constitutive transport vesicles from the TGN. PI3KIla might control clathrin-dependent sorting events at the TGN through a localized generation of PI3P at sites of clathrin-coated bud formation De Matteis et al. (2005)	PI3K	PI3K and COPII has been identified as regulators of LC3 lipidation vesicles using as membrane source the ERGIC compartment. ERGIC is required for autophagosome biogenesis *in vivo* by recruiting of ATG14, a critical step for the generation of PAS Ge et al. (2014)
It’s activated by Rab 1 to spatially regulate its kinase activity and phosphorylation of the COPII coat during protein transport from ER Wang et al. (2015)	CK1	Casein Kinase 1 mutants which are defective in ER-Golgi traffic also show autophagy dysregulation Wang et al. (2015)
A component of COPII with function on physical deformation of the ER membrane into vesicles and the selection of cargo molecules Miller and Barlowe (2010)	SEC24	Sec24 phosphorylation regulates autophagosomel number and its interaction with the C-terminus of ATG9, a transmembrane ATG protein involved in the initial step of PAS formation Davis et al. (2016)
SNARE proteins lie at the heart of the membrane fusion events in the secretory and endocytic pathways. Interactions between them provide the driving force for bringing membranes together, but also to contribute to the specificity of vesicle targeting Pelham (1999)	SNARES	VAMP7 (SNARE) is require for homotypic fusion of ATG16L1 with ATG12-ATG5 to LC3 lipidation. It’s have been implicated in ATG9A trafficking. Thus, autophagosome expansion/closure appears to proceed via a SNARE-dependent mechanism (Moreau et al. (2011); Shibutani and Yoshimori (2014)
Is a tethering factor for COP II vesicles that positively modulates the recruitment of Sec13/31 onto COPII vesicles. Plays a major role in regulating Rab activation and trafficking at the Golgi. Therefore, its Knockdown disrupts Golgi integrity and protein secretion Zhao et al. (2017)	TRAPPIII	TRAPPIII-like complex and TBC1D14 are needed for Rab1 activation to regulate constitutive ATG9 trafficking to the Golgi.During starvation, knockdown of TRAPPC8 subunits reduces the number of PAS, suggesting that it is required for early autophagy Lamb et al. (2016)
At the ER interacts with RINT-1 (Golgi-ER membrane traffic regulator), acting as an integral component of the RINT-1-containing ER tethering complex, in this way engage phosphoinositide metabolism to COPI-vesicles tethering, hence regulating Golgi to ER retrograde transport He et al. (2013)	UVRAG	Bind to a beclin-1, core component of Beclin 1-PI3KC3 complex involved in autophagosome nucleation. Autophagy caused the dissociation of UVRAG from the ER tether triggering ATG9 translocation for autophagosome formation He et al. (2013)
Is required for stabilization of Sec24D protein levels, maintenance of functional ERES, and efficient ER export in a manner dependent on binding to lipidated LC3. Deficient TECPR2 in cells display alterations in Sec24D abundance and ER export efficiency Stadel et al. (2015)	TECPR2	LC3 binds to TECPR2 and regulates autophagy. Both are required for autophagosome formation, possibly maintaining functional ERES. These results reveal that TECPR2 functions as molecular scaffold linking early secretion pathway and autophagy Behrends et al. (2010); Oz-Levi et al. (2012)
Participate in ER–Golgi traffic in normal growth and interact with Sec16A. ULK1 regulates the budding of COPII vesicles *in vitro*. *In vivo*, ULKmediated phosphorylation of SEC16A regulated the assembly of ERES and ER-to-Golgi trafficking without other autophagy proteins Sprangers and Rabouille (2015)	ULK 1/2	Known for their role in the induction and regulation of autophagy. Models have proposed that the active ULK1 directly phosphorylates Beclin- 1 at Ser 14 and activates the PI3K, VPS34 complex, to promote autophagy induction and maturation Russell et al. (2013)

Here, we address several cellular and molecular aspects that demonstrate how the early conventional secretory pathway and autophagy are mutually regulated, including the growing literature about the key regulatory clues under normal and stressful scenarios and their impact on cellular homeostasis maintenance.

### The early conventional secretory pathway between ER and golgi apparatus regulates autophagy

The two compartments of the early conventional secretory pathway, ER and Golgi apparatus, play a role in regulating autophagy at different levels, where the anterograde and retrograde pathways have complementary outcomes in this catabolic pathway. Concerning this, the coat protein complex II (COPII) and coat protein complex I (COPI) involved in ER-Golgi anterograde and retrograde Golgi to ER membrane transport, respectively (Bethune and Wieland, 2018) have specific contributions in how these two pathways control autophagy (Table 1).

### ER to golgi apparatus anterograde early secretory pathway as a regulator of autophagy

COPII complex is recruited hierarchically to the specific ER membrane spots called ER exit sites (ERES). Functional, proteomic, and cytological analyses have shown that autophagosomes are spatially, physically, and functionally linked to ERES (Graef et al., 2013; Lamb et al., 2013; Suzuki et al., 2013; Sanchez-Wandelmer et al., 2015). The COPII recruitment is initiated by the small G-protein Sar1, which is regulated by the exchange of GDP for GTP catalyzed by the ER membrane protein SEC12, which activates Sar1 and recruits the SEC23 and SEC24 heterodimer (Nakano et al., 1988; Nakano and Muramatsu, 1989; Barlowe et al., 1994; Mancias and Goldberg, 2008). SEC24 is the cargo binding protein (Miller et al., 2002) interacting with several different ER export motifs through various domains (Miller et al., 2003). The SEC23/SEC24 heterodimer also provides the platform for the recruitment of the outer layer of the COPII coat, a heterodimer formed by SEC13 and SEC31 (Sato and Nakano, 2007; Bisnett et al., 2020).

**FIGURE 1:**
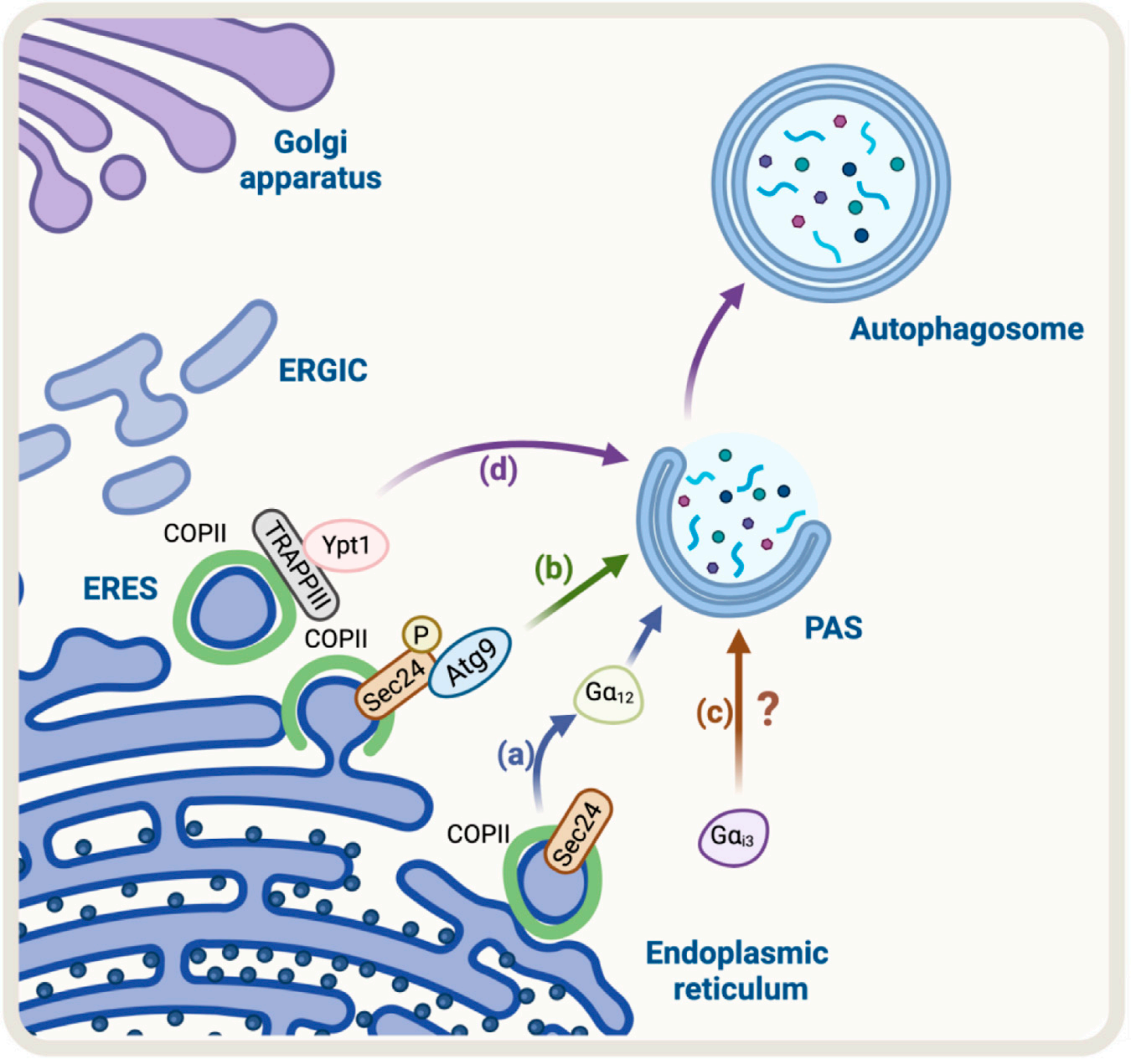
Schematic representation of the Regulators of ER-Golgi anterograde pathway and autophagy. **(A)** SEC24, a COPII protein, acts as a GEF activating the trimeric Gα_12_ GTPase to promote cargo export at the ERES. Gα_12_ activation is linked to autophagosome biogenesis. **(B)** Casein kinase 1 (Hrr25 in yeast) phosphorylates SEC24, promoting its interaction with Atg9 and autophagosome formation **(C)** Gα_i3_ regulates the initial steps of autophagosome formation. Still, the mechanism remains unknown **(D)** TRAPPIII, a transport complex that binds to the COPII complex, acts as a GEF for Rab GTPase Ypt1 (the yeast homolog of Rab1 in mammals). Ypt1 modulates PAS formation.

The first evidence that suggested the COPII complex was regulating autophagy was found in yeast. They observed that the genetic ablation of the SEC24, a component of the COPII complex, abolished autophagosome formation (Ishihara et al., 2001). Recently, SEC23 and its mammalian counterpart SEC24C have been implicated in autophagy as receptors, selecting ER membranes and cargo for degradation (Cui et al., 2019). Additionally, studies in mammalian cells have shown that the COPII complex is also required for autophagosome formation (Shima et al., 2019). The precise mechanism by which COPII regulates autophagosome formation is not fully understood, but it is thought to involve the regulation of the phosphatidylinositol 3-kinase (PI3K) complex, which is involved in the initiation of autophagosome formation (Ge et al., 2014). Overall, the evidence suggests that the COPII complex plays a crucial role in the regulation of autophagy by providing the necessary membrane for autophagosome formation and regulating the PI3K complex. Recently, using a vesicle–labeling system in fluorescence and immunoelectron microscopy, it has been confirmed that COPII vesicles play a role as a membrane source for autophagosomes (Shima et al., 2019). In agreement with this finding, treatment with FLI06, a drug that prevents the loading of cargo to the COPII complex and causes a blockage in general secretion at the ER, triggers a decrease in the levels of ATG13, a factor required for autophagosome formation, and a significant reduction in the number of autophagosomes, similar to the phenotype observed in non-starved cells (Karanasios et al., 2016). In addition, another piece of evidence refers to the role of SEC24, a protein that has been shown to participate in the signaling required for the exit of cargo from the ER, called autoregulation of ER export (AREX). SEC24 acts as a sensor of folded cargo by working as a guanine nucleotide exchange factor (GEF) to promote cargo export at the ERES by activating the trimeric Gα12 GTPase (Subramanian et al., 2019) (Figure 1A). Interestingly, it has been reported that the absence of SEC24 is also required to initiate autophagosomal biogenesis (Ishihara et al., 2001). Additionally, because the SEC13/SEC31 heterodimer is not necessary for autophagy, critical components of the COPII complexes suggest that vesicle formation and autophagosomal biogenesis share some important machinery but not all the structural players. This also opens the possibility that SEC24 is a common player in these two pathways. However, the mechanism of how SEC24 is recruited to the ER in one direction, or another is not yet understood. One possible mechanism might be related to SEC24 post-translational modifications. In this regard, several phosphorylation sites have been reported on SEC24; among them, phosphorylation on T324, T325, and T328 residues is required for autophagy. The phosphorylation of SEC24 by the casein kinase 1 (Hrr25, CK1 in mammalian cells) promotes its interaction with Atg9 and induces autophagosome formation (Davis et al., 2016) (Figure 1B), post-translational modifications that are not required for ER-to-Golgi anterograde transport in yeast. Alternatively, another explanation could be related to the function of the Gαi3 GTPase (Petiot et al., 1999). Gαi3 is located at the ER and Golgi apparatus and controls ADP-ribosylation factor 1 (Arf1) signaling, serving as a crossroad between signals controlled by the trimeric G proteins and the Arf family of monomeric GTPases to regulate early conventional anterograde pathway (Lo et al., 2015) (Figure 1C). Importantly, it has been reported that Gαi3 GTPase acts as a regulator of autophagy, controlling autophagosome biogenesis (Gohla et al., 2007), by a mechanism dependent on insulin signaling and nutrients (Gohla et al., 2007).

In addition, another protein mutually implicated is the transport protein particle complex (TRAPP), a tethering of COPII vesicles at the ER surface (Sacher et al., 2008). Mammalian cells have two forms of TRAPP complexes: TRAPPII and TRAPPIII (Yamasaki et al., 2009; Bassik et al., 2013; Wang et al., 2013). TRAPPIII plays a key role in the targeting and/or fusion of ER-to-Golgi transport vesicles with their acceptor compartment (Sacher et al., 1998; Scrivens et al., 2011). TRAPPIII, specifically its TRAPPC12 subunit, interacts with SEC13/SEC31, modulating positively the SEC13/SEC31 tetramer assembly in COPII-positive vesicles (Zhao et al., 2017). TRAPPC12 localizes to ERES and its deletion causes ER-to-Golgi transport delay (Zhao et al., 2017). Moreover, it has been proposed that TRAPPIII acts as a GEF of the Rab GTPase Ypt1, which is involved in the membrane tethering during the formation of the PAS. Ablation of Bet1, a component of TRAPPIII, can disrupt autophagy (Tan et al., 2013). These findings confirm that COPII and TRAPPIII complexes are also involved in autophagosome formation (Figure 1D).

ERES plays a role in autophagosome formation by mediating contact sites with ERGIC through TMED9 and SEC12 (Li et al., 2021). ERGIC-ERES contact promotes the formation of the COPII vesicle, a precursor of the autophagosome derived from ERGIC. This contact differs both physically and functionally from the TFG-mediated ERGIC-ERES interaction involved in the early secretory pathway (Witte et al., 2011; Johnson et al., 2015; Hanna et al., 2017). TMED9 RNAi, which inhibits SEC12 recruitment and autophagosomes formation, does not affect protein secretion (Li et al., 2022). This provides a mechanistic insight by which ERES can switch between autophagosomes formation and ER-to-Golgi cargo transport.

TMEM39A is an ER-localized transmembrane protein that regulates autophagy by affecting the distribution and levels of PI4P. It interacts with the ER-localized PI4P phosphatase SAC1, an integral membrane protein that cycles between the ER and Golgi (Del Bel and Brill, 2018), and promotes its ER-to-Golgi transport via COPII SEC23/SEC24 subunits. The knock-down of TMEM39A leads to SAC1 retention in the ER, resulting in increased autophagosome formation (Miao et al., 2020).

Altogether, these findings provide strong evidence for the existence of a close functional and interorganelle coordination between the conventional early secretory pathway and autophagy, which requires further investigation in mammals.

### Golgi-to-ER retrograde early secretory pathway as a regulator of autophagy

**FIGURE 2:**
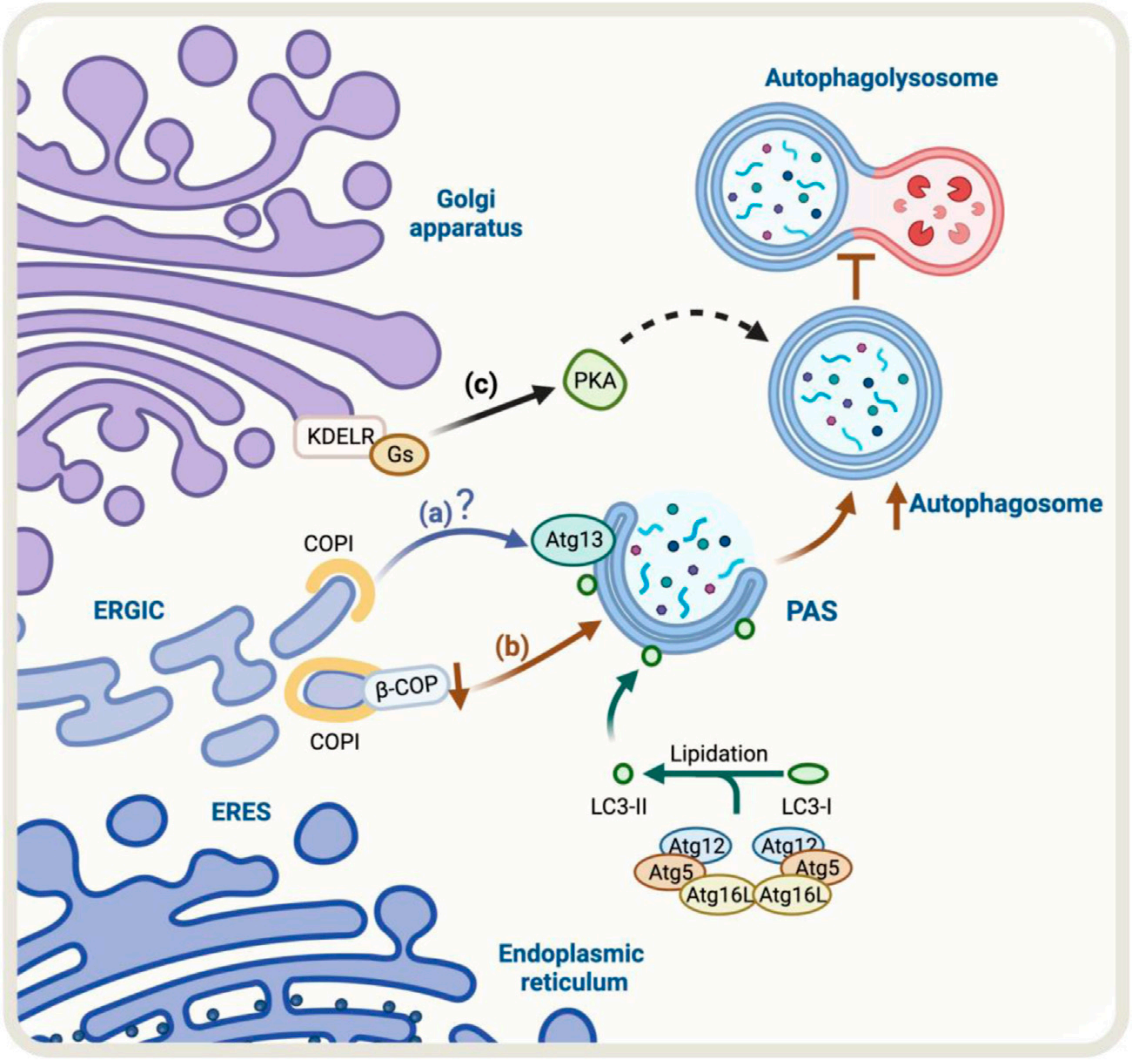
Schematic representation of the regulators of Golgi-to-ER retrograde pathway and autophagy. **(A)** Under starvation conditions, COPI localizes at the ERGIC. COPI-mediated trafficking may recruit Atg13, however, the molecular mechanism is still unclear (blue arrow). **(B)** The knock-down of β-COP, a protein of the COPI complex, results in an accumulation of autophagosomal structures. Still, it also partially hinders the fusion of the autophagosome with the lysosome and the degradation and recycling process (brown arrows) **(C)** KDEL/Gs signaling pathway, which includes PKA activation, may be involved in the upregulation of autophagy (black arrows). The green arrow represents the process of lipidation of LC3 protein by Atgs proteins that is necessary for substrate recognition and autophagosome formation.

The Golgi-to-ER retrograde early secretory pathway has been shown to play a role in regulating autophagy (Tapia et al., 2019; Bustamante et al., 2020). In this context, a crucial protein complex implicated in this retrograde pathway is the coat protein complex COPI, responsible for forming a coat around transport vesicles, which helps to protect the contents of the vesicles during transport and directs them to specific destinations. This complex consists of seven core subunits α-COP, β′-COP, ε-COP, β-COP, δ-COP, γ-COP, and ζ-COP, the coatomer. The heptameric cytosolic coatomer is recruited to the Golgi membrane by the small GTPase Arf1 together with other Arf family members to form the COPI coat (Hara-Kuge et al., 1994; Popoff et al., 2011). Additionally, COPI has been linked with the autophagy pathway (Karanasios et al., 2016). In fact, under starvation conditions, a pool of COPI is relocated to the ER-Golgi intermediate compartment (ERGIC), a subcompartment of the ER located between the cis-Golgi and the medial-Golgi, which is thought to play a role in regulating autophagy (Razi et al., 2009; Karanasios et al., 2016). In this context, during starvation, a pool of COPI colocalizes with ERGIC53, a marker of the ERGIC compartment (Razi et al., 2009). Additionally, BFA, a fungal metabolite that blocks COPI-dependent trafficking (Lippincott-Schwartz et al., 1989), decreases autophagy induced by amino-acid starvation (Karanasios et al., 2016). When cells are treated with BFA, it interferes with the recruitment of Atg13 to autophagosomes, a component of the ULK1 complex required for the initiation of autophagy, where its recruitment to autophagosomes is necessary for the proper initiation of autophagy (Karanasios et al., 2016) (Figure 2A).

The lipidation of LC3 to form LC3-II is an obligatory step for the expansion of the autophagosomal membrane (Tanida et al., 2008), a stage in which COPI participates (Karanasios et al., 2016). In contrast, COPI is not necessary for the steps upstream of LC3 lipidation, such as nucleation (Ge et al., 2013). When COPI function is lost, it leads to fragmentation of the Golgi apparatus, accumulation of immature autophagosomes, and a decrease in the overall process of autophagy (Shtutman and Roninson, 2011). Moreover, the reduction or elimination of specific components of COPI, such as β-COP, leads to an increase in the formation of autophagosomes. However, this also hinders the fusion of these autophagosomes with lysosomes and the subsequent degradation and recycling process (Claerhout et al., 2012) (Figure 2B). Although the COPI complex is required for autophagy, the precise role of COPI in this pathway is not yet fully understood, and further research is needed to determine its specific mechanisms of involvement in this process.

The Golgi apparatus can maintain and recover its normal composition and morphology upon cellular stress, such as changes in transport rates to and from Golgi (Mironov et al., 2001; Trucco et al., 2004). In this regard, the Golgi reassembly stacking protein of 55 kDa (GRASP55) is required for Golgi cisternae stacking, ribbon linking, and mitotic fragmentation (Shorter et al., 1999; Duran et al., 2008; Feinstein and Linstedt, 2008; Xiang and Wang, 2010). Although GRASP55 is viewed as a Golgi protein, it is also found at the ER-Golgi interface (Piao et al., 2017). GRASP55 is not strictly required for conventional protein secretion (Duran et al., 2008; Feinstein and Linstedt, 2008) but GRASP55 knock-down increases protein secretion (Xiang et al., 2013). On the other hand, GRASP55 knockdown and CRISPR-Cas9 deletion in HEK293T cells enhance LC3 puncta formation, indicating that GRASP55, required for ER-Golgi organization, restricts autophagosome formation (Liu et al., 2021).

One homeostatic mechanism that has been reported is based on the signaling properties of the KDEL receptor (KDELR) (Pulvirenti et al., 2008; Giannotta et al., 2012; Cancino et al., 2014). The KDELR shares functional properties with G protein-coupled receptors (Giannotta et al., 2012; Solis et al., 2017) and regulates transport out from the Golgi (Pulvirenti et al., 2008; Giannotta et al., 2012; Cancino et al., 2014). KDELR binds to and recycles chaperones that escape the ER to the Golgi using the Golgi-to-ER retrograde pathway in yeast (Semenza et al., 1990) and mammals (Lewis and Pelham, 1990). In this context, KDELR signaling in the early conventional secretory pathway controls trafficking through downstream signaling pathways that involve two heterotrimeric Gα proteins, Gs and Gq (Giannotta et al., 2012; Cancino et al., 2014). The activation of the KDELR/Gs signaling pathway plays an important role in maintaining the homeostasis of the Golgi apparatus. This signaling cascade involves the activation of protein kinase A (PKA) which phosphorylates the COPI subunits, increasing the dynamics of the COPI coat complex (Cancino et al., 2014). The activation of the KDELR/Gs signaling pathway has been shown to increase autophagy, by increasing the formation of autophagosomes and their fusion with lysosomes. This is a new function for KDELR (Tapia et al., 2019) (Figure 2C). However, it is not yet clear whether the PKA-dependent COPI phosphorylation is required for this response to take place, more research is needed to understand the underlying mechanism. Despite this, studies have shown that the KDELR pathway plays a role in promoting autophagy under stress conditions, making it a new and exciting area of research that might lead to a better understanding and regulation of the autophagic process. The accumulation of misfolded proteins related to neurodegenerative diseases increases KDELR mRNA. Additionally, overexpression of KDELR, but not the signaling-defective KDELR-D193N mutant, has been shown to induce autophagy to promote the clearance of protein associated with diseases, such as superoxide dismutase (SOD1), Parkinson’s disease-associated A53T alpha-synuclein, and Huntington’s disease-related expanded huntingtin (Wang et al., 2011). This response is not controlled by, but rather by the mitogen extracellular kinase 1 (MEK1). Therefore, this KDELR pathway seems to involve more than one signaling pathway.

In addition to COPI and KDELR-dependent signaling, another protein that regulates Golgi-to-ER retrograde pathway is PSMD14, a subunit of the 19S regulatory particle of the proteasome with deubiquitinating (DUB) enzyme activity (Bustamante et al., 2020). Importantly, pharmacological inhibition of PSMD14 with Capzimin (CZM) has been shown to act as a potent blocker of autophagy by a mechanism related to the decay in the Golgi-to-ER retrograde transport. Additionally, research suggests that PSMD14’s DUB activity plays non-canonical roles not coupled to the translocation of substrates into the core of the 20S proteasome (Bustamante et al., 2023). This opens the possibility that during stress conditions, the upregulation of autophagy may require an active Golgi-to-ER retrograde transport. Further studies such as kinetic studies to explore the speed of vesicle transport labeled with specific cargoes of the Golgi-to-ER retrograde transport could provide more insight into the role of PSMD14 in this process.

### Autophagy controls the early conventional secretory pathway

Autophagy adds another level of communication that includes its interplay with the conventional secretory pathway, and in recent years, several new findings confirm these functional connections. Upon starvation, the *de novo* formation of autophagosomes is upregulated and is driven by conserved machinery composed mostly of cytosolic complexes that are sequentially and hierarchically recruited to the ER (Ohsumi, 2001; Suzuki et al., 2007). Autophagy is a complex multi-step process that involves various proteins and molecular machinery. It is regulated by cytosolic proteins that control the initiation of autophagosome formation, such as the Ser/Thr kinase Unc-51-like kinase-1 complex (ULK1; Atg1-complex in yeasts) (Scott et al., 1996; Neufeld, 2007), the transmembrane protein Atg9 which is required for phagophore elongation (Zhou et al., 2017), and the class III PI 3-kinase complexes C1 and C2 (Ma et al., 2017). These complexes are differentiated based on the composition of their subunits and their specific functions. The PI3K3C-C1 complex, which includes the catalytic subunit Vps34 and the regulatory subunit Beclin-1, plays a crucial role in the initiation of autophagosome formation by inducing the production of phosphatidylinositol 3-phosphate (PtdIns3P) (Axe et al., 2008) that recruits other necessary proteins to the site of PAS formation such as WIPI 1-4, Atg8/LC3 family, and the Atg12 system (Simonsen and Tooze, 2009; Itakura and Mizushima, 2010). The PI3K3C-C2 complex, consisting of the catalytic subunit Vps34, the regulatory subunit Beclin-1, and the UV radiation resistance-associated gene protein (UVRAG), is involved in the maturation of autophagosomes. The inclusion of UVRAG instead of Atg14 in the PI3K3C-C1 complex leads to the formation of PI3K3C-C2, which contributes to autophagosome maturation (Liang et al., 2008). Moreover, growing evidence indicates that a functional integration between the early conventional secretory pathway and autophagy plays an important role in maintaining cellular homeostasis, sharing important molecular players (Lamb et al., 2013). Interestingly, the connection between these pathways is not limited to shared molecular players; structural and regulatory autophagy proteins also depend on membrane transport to function effectively (Mari et al., 2010; Kakuta et al., 2012; Orsi et al., 2012; Imai et al., 2016; Lamb et al., 2016; Lamb et al., 2017; Zhou et al., 2017)

**FIGURE 3:**
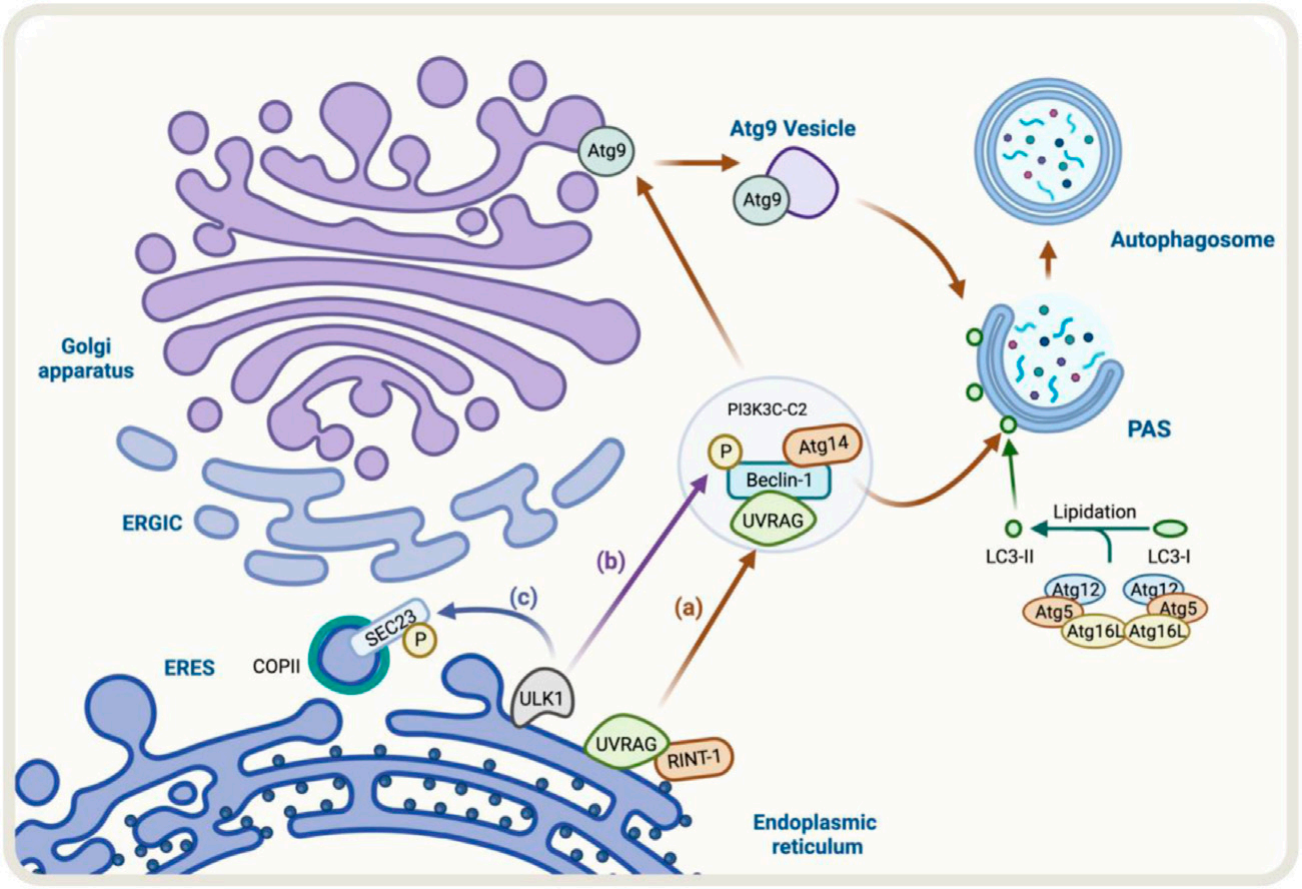
Illustration of the regulators of autophagy pathway controlling the early conventional secretory pathway. **(A)** UVRAG binds to the PtdIns3P-enriched membranes at the ER where it interacts with RINT-1. During autophagy induction, UVRAG dissociates from the ER to become part of the complex PI3K3C-C2 (for simplicity, only the Beclin-1, Atg14, and UVRAG proteins are shown). The dissociation of UVRAG from the ER induces the translocation of the Atg9 vesicles, necessary to promote autophagosome formation (brown arrows) **(B)** The ULK1 complex can phosphorylate Beclin-1 inducing autophagy (purple arrow). **(C)** Additionally, ULK1 phosphorylates SEC16A promoting anterograde transport (blue arrow).

The formation of autophagosomes begins at multiple PtdIns3P-enriched cup-like subdomains of the ER, a structure called PAS (Axe et al., 2008). Studies have demonstrated that COPII components are recruited to the ERGIC in a phosphatidylinositol kinase class III (PI3K)-dependent manner during autophagy (Ge et al., 2014). Concerning this, UVRAG is a PtdIns3P-binding protein. During autophagy induction, UVRAG coordinates Golgi-to-ER retrograde transport including the cargo ATG9A, by differential interactions with the ER tether and the BECLIN-1 complex (He et al., 2013) (Figure 3A). At the ER, UVRAG interacts with RINT-1 (Golgi-ER membrane traffic regulator); this way engages phosphoinositide metabolism to COPI-vesicle tethering, hence regulating Golgi-to-ER retrograde transport. Moreover, during autophagy induction, UVRAG dissociates from the ER tether, triggering ATG9A translocation for autophagosome formation (He et al., 2013) (Figure 3B).

ULK1/2 is a serine/threonine protein kinase that regulates autophagy (Jung et al., 2009; Joo et al., 2016), a kinase that also has been shown to play a role in ER-to-Golgi anterograde pathway (Joo et al., 2016). By proteomics approach, SEC16A (molecular scaffold which participates in the organization of ERES), was identified as a ULK1/2 interaction partner. ULK1 activity regulates the formation of COPII vesicles *in vitro*. *In vivo*, ULK1-phosphorylates SEC16A regulates the assembly of ERES and, consequently, ER-to-Golgi anterograde transport (Sprangers and Rabouille, 2015) and does not require other autophagy proteins (Joo et al., 2016). ULK1 can also phosphorylate SEC23, an essential protein of the COPII complex, phosphorylation that alters the morphology of ERES reducing global cellular secretion (Gan et al., 2017) (Figure 3C).

While inhibition of COPII transport impairs starvation-induced autophagy (Zoppino et al., 2010; Graef et al., 2013), the role of ULK1/2-mediated phosphorylation of SEC16A in autophagy is unknown. Because SEC16A is a target of several kinases (Farhan et al., 2010; Zacharogianni et al., 2011; Tillmann et al., 2015), its posttranslational modification may provide a means of adjusting the regulation of COPII transport with the availability of nutrients (Farhan et al., 2017).

Collectively, these findings demonstrated that autophagy and membrane transport systems are closely related, mutually dependent, and functionally coordinated. However, the extent of this functional integration is poorly understood and will require deep investigation to establish its principles and molecular architecture.

### Interorganelle crosstalk between the early conventional secretory pathway and autophagy under stressful conditions

The early conventional secretory pathway changes for cell survival in response to stress to maintain homeostasis and prevent damage to the cell. When the cell experiences stress, it triggers signaling pathways that alter the function of the secretory pathway, including the ER and the Golgi apparatus. These changes can include changes in protein folding and trafficking, as well as the activation of autophagy. Ultimately, these changes in the early secretory pathway aim to restore cellular balance and promote adaptation mechanisms for cell survival (Ding et al., 2007; Kroemer et al., 2010; Murrow and Debnath, 2013).

**FIGURE 4:**
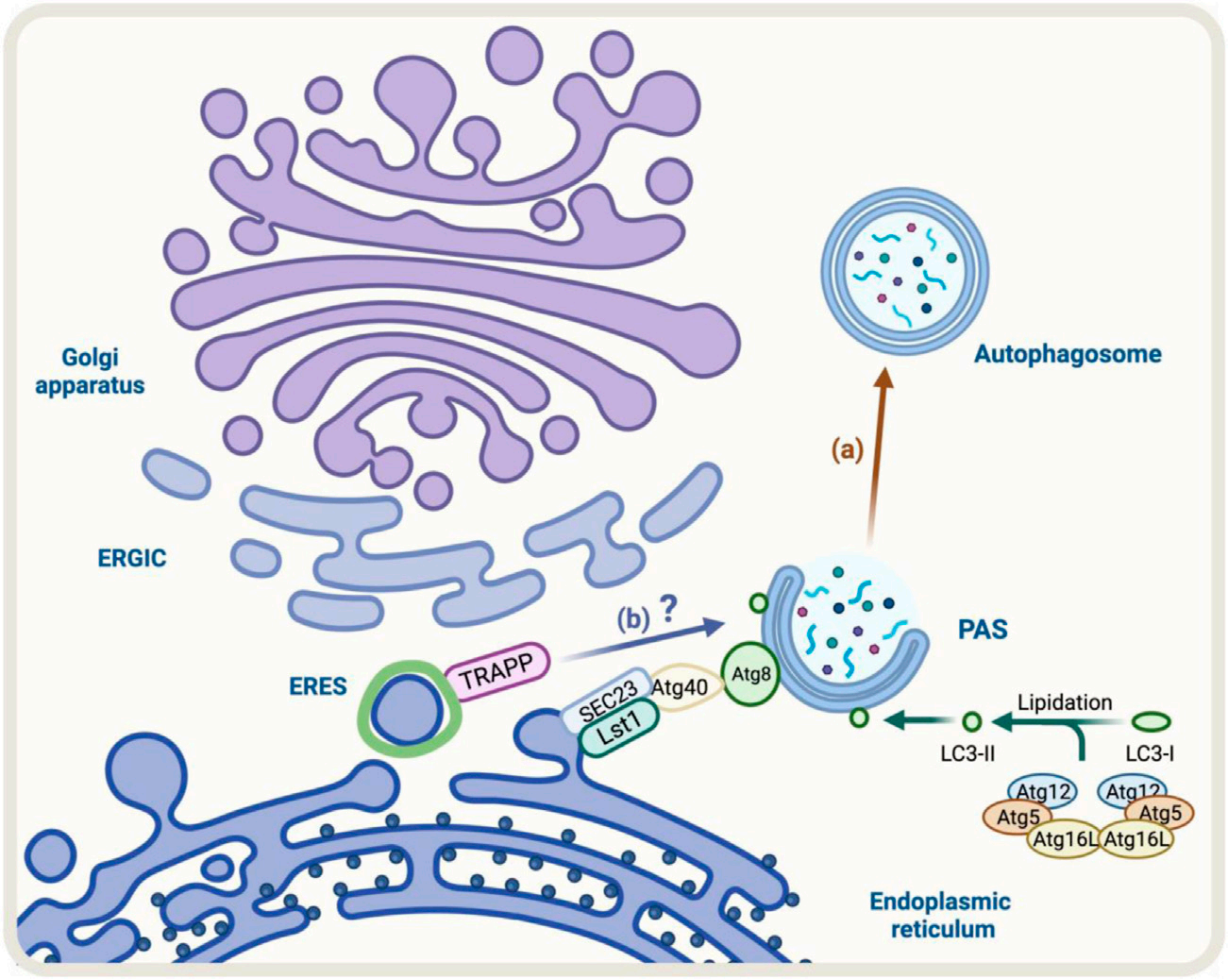
Diagram representation of the regulators of autophagy and early conventional secretory pathway under stress. **(A)** During ER-phagy, components of COPII complex such as Lst1/SEC24C-SEC23 can interact with Atg40, a specific ER-phagy receptor, which binds to Atg8/LC3 allowing the degradation of the ER by autophagy (brown arrow) **(B)** TRAPP components respond to Golgi stress with impact on autophagy, however, the molecular mechanism is not fully resolved (blue arrow).

A good example of these cellular adaptations is the ER-phagy process that occurs under ER stress (Song et al., 2018). The ER-phagy process is a cellular adaptation that occurs under ER stress and involves the degradation of the endoplasmic reticulum (ER) by autophagy. In yeast, some components like Lst1/SEC24C-SEC23 that belong to the COPII complex play a role in ER degradation through ER-phagy. This is because they can interact with Atg40, a specific ER-phagy receptor, which binds to Atg8/LC3 (Kercsmar et al., 1988; Cui et al., 2019) (Figure 4A). Under stress, the COPII machinery may decrease to conserve energy, while autophagy may increase to recycle cellular components and provide energy. Altogether, the increase in Golgi-to-ER retrograde transport and formation of autophagosomes indicate strong communication between the conventional secretory pathway and autophagy during ER stress.

The impact of osmotic stress on the early conventional secretory pathway has been studied. The data show that in a hypertonic treatment, the ER to Golgi transport is inhibited (Docherty and Snider, 1991). Conversely, a hypotonic treatment promotes the tubulation of the Golgi membranes (Lee and Linstedt, 1999). Similarly, hyperosmotic conditions induce the fragmentation and redistribution of the Golgi back to the ER, as seen with Brefeldin A (BFA) treatment (Lippincott-Schwartz et al., 1990; Sciaky et al., 1997; Lee and Linstedt, 1999). In contrast, hypo-osmotic conditions result in a reduction in the number of export sites on the ER, leading to less trafficking from the ER to the Golgi apparatus (Lee and Linstedt, 1999).

Regulation of intracellular trafficking involves Rab proteins, which are small GTPases that control the formation and movement of vesicles between intracellular compartments (Zerial and McBride, 2001; Grosshans et al., 2006). Rab1 and Rab6 have been found to play a role in the anterograde and retrograde traffic between the ER and Golgi apparatus, respectively (Ortiz Sandoval and Simmen, 2012). In yeast, Rab1 has been linked to the regulation of the UPR (Tsvetanova et al., 2012). However, in hippocampal cells during UPR, the levels of Rab1 decrease along with the cell viability. In contrast, Rab6 and UPR-associated protein increase in levels, suggesting a connection between vesicle trafficking and ER homeostasis. In the context of Alzheimer’s disease, ER stress causes neuronal death. The stress signals propagate from the ER to Golgi, affecting the transport and processing of AD-related proteins such as β-amyloid precursor protein (Suga et al., 2009). Regarding this, inhibition of the Golgi-to-ER retrograde pathway by silencing PSMD14 leads to a dramatic increase in the levels of the β-amyloid precursor protein (Bustamante et al., 2020). Additionally, ER stress increases the expression of the ER-Golgi SNARE protein Syntaxin5 (Syx5), which is crucial for the fusion of vesicles with early stations in the conventional secretory pathway, leading to a reduction in the secretion of amyloid β peptide (Suga et al., 2015). In this context, in the primary culture of rat hippocampal neurons, ER stress leads to an increase in the expression of Syx5 and Bet1 (another SNARE protein complex involved in ER-to-Golgi transport) through the *novo* synthesis (Donkervoort et al., 2021). The impact of these changes in SNARE proteins on autophagy during stress conditions is still unknown, but it has been suggested as a possibility. In yeast, Ufe1, a Qa/t-SNARE localized in the ER, participates in autophagy. During starvation, it is exported from the ER to intracellular locations containing autophagy markers Atg8 and Atg9, where it interacts with non-ER SNARE involved in autophagosome formation (Lemus et al., 2016). Data suggests that Ufe1 reaches autophagosome formation sites via specific COPII vesicles, connecting ER stress changes to autophagy.

Another factor that responds to cellular stress is TRAPPC13, a subunit of the TRAPP complex involved in ER-to-Golgi and intra-Golgi transport (Barrowman et al., 2010). During Golgi stress induced by BFA, the absence of TRAPPC13 protects cells from death caused by BFA treatment and is correlated with reduced autophagy, suggesting TRAPPC13’s role in autophagy and autophagy’s role in cell death due to prolonged Golgi stress (Ramirez-Peinado et al., 2017) (Figure 4B). Other TRAPP complex subunits (TRAPPC12, TRAPPC11, TRAPPC9, and TRAPPC3) also behave similarly to TRAPPC13 against BFA-induced toxicity, indicating multiple TRAPP complex components play a role in the Golgi stress response and autophagy (Barrowman et al., 2010).

STING1, a transmembrane protein that cycles between Golgi and ER via a COPI-dependent mechanism, is another example (Burdette et al., 2011; Mukai et al., 2021). During stress from pathogen infection (bacteria and viruses), STING1 positively regulates autophagy (Watson et al., 2012; Collins et al., 2015) and is reported to interact and co-localize with LC3 and ATG9 (Saitoh et al., 2009). These findings suggest different stress conditions can alter the normal functioning of early secretory pathway proteins, which in turn could directly influence the normal development of autophagy. An excellent example is the UVRAG-RINT-1 interaction. Under non-stressful scenarios, these proteins play an important role in Golgi-to-ER retrograde transport (He et al., 2013). However, under stress by nutritional privation, UVRAG dissociates from RINT-1, instead interacting with Beclin-1 to promote autophagosome biogenesis (He et al., 2013). Intriguingly, lack of beclin-1 can result in an enlarged Golgi apparatus with higher levels of PI4P and proteins that normally traffics in the Golgi-to-ER retrograde transport, such as β′-COP (Bork et al., 2022), suggesting beclin-1 is also a key protein in the early conventional secretory pathway.

Despite our understanding of these cellular adaptations, there is still a need for further research to uncover the full extent of changes that occur under various stress conditions. This will help us gain a deeper understanding of the mechanisms involved and develop more effective treatments in the future to treat diseases.

### Future directions and concluding remarks

To understand cell survival strategies, particularly under stress, it's crucial to unlocking the connection between early conventional secretion and autophagy. The relationship between these pathways has important implications for cellular metabolism and helps us understand how cells adapt and survive in changing conditions. A comprehensive view of organelles such as lipid droplets, mitochondria, and lysosomes will enhance our understanding of the interactions between these pathways and their response to stressors. It’s crucial to expand our understanding of how cells respond to lipid imbalance and fatty acid (FAs) overload, given the recent global dietary changes. Cells respond to high energy demands by moving fatty acids to mitochondria for ATP production. As a result, intracellular membranes create MCSs to coordinate inter-organelle communication, enabling the cellular plasticity events needed for adaptation and survival. However, regulation of MCSs during protein secretion and the roles of autophagy and membrane transport machinery is still unknown. Gaining an understanding of the crosstalk between pathways involved in intracellular membrane networks will shed light on how cells coordinate stress responses, especially during periods of high protein secretion demand. The discovery of TIDeRS (traffic-induced degradation response for secretion) highlights the significance of inter-organelle coordination under different nutrient conditions, with a focus on the connection between lipid droplets and protein secretion. Finally, the mutual regulation between ER-Golgi transport, Golgi-to-ER retrograde pathway, autophagy, and its impact on each other raises many questions and offers future opportunities for developing therapeutic interventions in biomedical fields.

## Abbreviations

ER, endoplasmic reticulum; ERES, ER exit sites; ER Golgi intermediate 19 compartment, ERGIC; BFA, Brefeldin A; KDELR, KDEL receptor; PAS, pre-autophagosomal 20 structure; LD, lipid droplets; PKA, protein kinase A; PM, plasma membrane; TIDeRS, traffic-21 induced degradation response for secretion.
